# Between-Day Reliability of Visuomotor Response Times Under Stroboscopic Conditions Varying in Difficulty

**DOI:** 10.1177/00315125251382784

**Published:** 2025-10-03

**Authors:** Daniel Büchel, Thorben Hülsdünker, Jochen Baumeister

**Affiliations:** 1 Exercise Science & Neuroscience Unit, Department of Exercise & Health, 26578 Paderborn University, Paderborn, Germany; 2 Department of Exercise and Sport Science, LUNEX, Differdange, Luxembourg; 3 Luxembourg Health & Sport Sciences Research Institute (LHSSRI), Differdange, Luxembourg

**Keywords:** peak performance, attention/ distraction, visual perception, new tests/ batteries, motor skills & ergonomics, exercise response, athlete psychological preparation

## Abstract

Stroboscopic training (ST) effectively enhances visuomotor performance in athletes, yet the dose-response relationship between ST difficulty and performance remains unclear. This study investigated the influence of ST difficulty on visuomotor response times (RTs) and assessed the reliability of RTs under stroboscopic vision.

Twenty-two healthy young individuals performed a visuomotor response task on three separate days, responding to light-based stimuli under normal and stroboscopic vision at three difficulty levels (FAST = 6 Hz; MEDIUM = 4 Hz; SLOW = 2.25 Hz). Intraclass Correlation Coefficients (ICC) and Coefficients of Variation (CoV) assessed relative and absolute reliability. Repeated measures ANOVAs examined the effects of difficulty (NORMAL, FAST, MEDIUM, SLOW) and session day (I, II, III) on RTs.

Results showed significantly slower RTs at higher difficulty levels (*p* < .001), while session day had no significant effect. Reliability analysis revealed good to excellent relative reliability for NORMAL, FAST, and MEDIUM conditions, but moderate reliability for SLOW. Absolute reliability was acceptable across all conditions (<5%).

These results suggest a dose-response relationship between ST difficulty and RTs. The inter-individual variability in RTs under stroboscopic vision highlights the need for individualized ST difficulties. The high reliability scores suggest that performance changes following ST stem from functional adaptations rather than habituation.

## Introduction

Athletes in invasion, racket, and combat sports compete in constantly changing environments. These are often called open-skill sports because they require athletes to continuously process environmental information and adapt their movements (Knapp, 1967). Among the sensory information processed, visual cues are vital and allow for the encoding of decisive information such as object speeds, trajectories or distances (Buscemi et al., 2024). Consequently, the ability to effectively perceive and process visual cues is a cornerstone of sport-specific demands and decisive for sports performance (Laby et al., 2011).

Given the importance of vision in sports, training the ability to detect and integrate visual information appears to be a promising approach for enhancing sports performance (Lochhead et al., 2024). Here, the application of stroboscopic vision has become a prominent method to train the visual system (Wilkins & Appelbaum, 2020). Stroboscopic training reduces the quantity of visual information through a rhythmic, but adjustable occlusion of the visual input. Therefore, stroboscopic visual conditions force the visual system to process the available information more efficiently. Portable technologies such as stroboscopic glasses can be easily implemented into sports-specific training during tasks that require visuomotor abilities (Hülsdünker et al., 2019; 2021b). While several studies report effects on the behavioral level, it has also been shown that stroboscopic training modulates the neural encoding of information in the visual cortex. For instance, visual evoked potentials appear faster after stroboscopic training interventions, suggesting that the brain responds to visual cues more quickly (Hülsdünker et al., 2021a; Zwierko et al., 2024).

Although there is increasing evidence supporting the positive effect of stroboscopic vision on visuomotor performance, little is known about the exact prescription of stroboscopic training interventions. While the acute exposure to stroboscopic vision delays reaction times (Hülsdünker et al., 2023) and impairs sport-specific performance (Beavan et al., 2021), less is known about the acute effects of varying stroboscopic settings. In particular, it remains largely unclear if there is a dose-response relationship between performance and difficulty settings in stroboscopic training (Das et al., 2023). In stroboscopic training, the difficulty can be modulated by the frequency (number of shutters per second) and the duty cycle (relative proportion of opaque states during one clear-opaque cycle in %). These two factors determine the amount of visual information occluded over time, where difficulty increases with lower frequencies and longer duty cycles. Previous research has shown that response times in computer-based tests significantly decrease for lower frequencies, indicating an exponential dose-response-relationship, where performance collapses towards slower frequencies (Bennett et al., 2018). So, modulating stroboscopic frequencies appears to be a crucial factor in adjusting the difficulty level of stroboscopic training. However, it remains unclear if the results obtained by Bennett et al. (2018) in computer-based assessments can also be expected in more dynamic and field-based visuo-motor tasks. Accordingly, further research is needed to determine how different stroboscopic settings affect performance in sport-related tasks.

Additionally, commercially available stroboscopic eyewear is generally not synchronized with the timing of visual information presentation. Therefore, the relative timing between stimulus presentation and the shutter of the glasses varies from trial to trial and strobe glasses add a degree of randomness to task performance. Particularly for lower frequencies, this can increase the variability of task performance and reduce the reliability of the performance impairment. Understanding the reliability of performance under stroboscopic vision is an essential measure to anticipate the long-term effects of stroboscopic training, since the challenge induced by the goggles should be predictable (Brinkman et al., 2021). The reliability of task performance also contributes to the identification of practice effects induced by habituation to stroboscopic eyewear, a concern that has been raised but not quantified in previous studies (Ellison et al., 2020). However, no study has yet assessed how stroboscopic difficulty affects visuomotor performance while also considering task reliability.

Despite its growing popularity, two critical questions about stroboscopic training remain unanswered. First, the ideal dose is unknown; it is unclear how manipulating difficulty settings like frequency and duty cycle impacts performance. Second, the reliability of performance under stroboscopic vision is questionable, as the random timing of the eyewear’s shutters may introduce unpredictable variability. Without understanding this dose-response relationship and its reliability, prescribing effective training protocols is difficult. Therefore, this study aimed to identify the intra-individual effects of varying difficulties of stroboscopic eyewear on visuomotor performance. Therefore, more research is needed to understand how different stroboscopic settings affect sport-related performance. According to Bennett et al. (2018), we expected that more difficult visual conditions in terms of slower frequencies and longer duty cycles increase response times (Bennett et al., 2018). Further, between-day reliability was determined for each stroboscopic difficulty level. It was hypothesized that with lower frequencies and longer duty cycles, the task reliability drops due to a higher degree of randomness between stimulus presentation and the shutter of the glasses.

## Methods

### Participants

Bennett et al. (2018) reported a partial eta^2^ of .2 when comparing different strobe frequencies in a multiple object tracking task in 18 young adults. To achieve .9 statistical power at an alpha level of .05 using, a required sample size of 13 was calculated for this study using GPower, Version 3.1 (Faul et al., 2007). Accounting for potential attrition, 21 participants (7 females, age: 22.1 ± 1.6 years; height: 177.0 ± 10.7 cm; mass: 73.3 ± 10.9 kg) participated in the study. All individuals were physically active sports science students (6.8 ± 2.9 h of exercise/week) and were voluntarily recruited from the affiliated university of the first author based on convenience-sampling. Therefore, they represent a sample of physically active young adults. At the time of measurement, all participants were physically fit and free from any acute or chronic conditions affecting visuomotor performance. Further, individuals suffering with epilepsy or a history of migraine, dizziness or concussion symptoms in the last six months were excluded from the investigation (Das et al., 2023). Prior to participation, all individuals provided written informed consent. The study was approved by the ethics committee of the first author´s affiliated university (Nr.53/2024).

### Procedures

**Figure 1.**
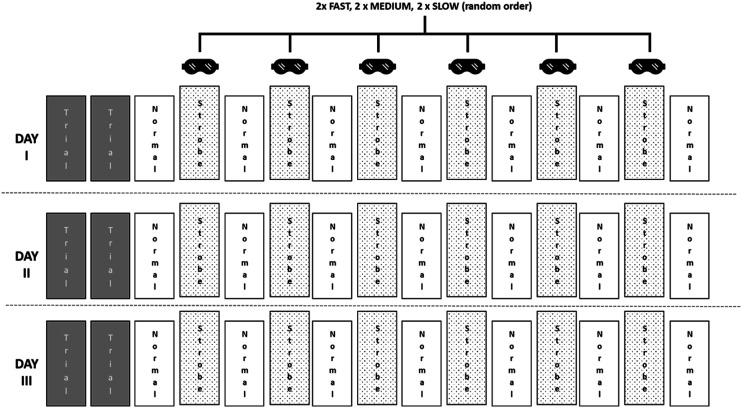
Schematic Overview of the Experimental Design. Participants Performed the Same Task once in Three Consecutive Weeks. For the Testing of Visuomotor Response Time Under Stroboscopic Vision, Three Different Stroboscopic Frequencies Were Utilized (FAST, 6 Hz, Duty Cycle 40 %; MEDIUM, 4 Hz, Duty Cycle 60 %; SLOW, 2.25 Hz, Duty Cycle 77 %). In Total, 15 Trials Were Performed per Session, and each Second Trial was a Normal Trial Without Stroboscopic Ayewear. For each Trial, 15 Lights had to be Deactivated

Each participant completed three laboratory sessions, each separated by one week. To minimize the potential impact of diurnal variation on visual performance, all sessions were conducted at similar times (±1 hour). Before each measurement, individuals filled out a short questionnaire assessing perceived sleep quality, perceived physical readiness to train, and mental readiness to train using a visual analogue scale (VAS) from 1 to 10 (only whole integers). The questionnaire aimed to identify participants with significant variations in well-being across days for exclusion from analysis. Physical and mental readiness to train were defined as the degree of how ready the participant felt physically and mentally to complete the test. Such single-item questionnaires have been validated in previous studies focusing on sleep quality (Badahdah et al., 2024) or physical readiness to train (Ten Haaf et al., 2017). After providing information on subjective well-being, participants started the experimental procedure and performed two familiarization trials of the visuomotor reaction task, once with and once without stroboscopic eyewear (Strobe Glasses Pro, Senaptec, Beaverton, United States). Three different stroboscopic difficulties were utilized, a fast frequency (FAST; 6 Hz: 100 ms clear, 67 ms opaque; duty cycle: 40 %), a medium frequency (MEDIUM; 4 Hz; 100 ms clear,&150 ms opaque; duty cycle: 60 %) and a slow frequency (SLOW; 2.25 Hz; 100 ms clear, 344 ms opaque; duty cycle: 77 %). Each stroboscopic condition was performed twice per session in a randomized order. Between each stroboscopic condition, a trial without stroboscopic eyewear was performed (NORMAL) and the rest interval between trials was set to 90 seconds. In total, 15 repetitions were performed per trial and the median visuomotor response time (RT) for each trial was extracted. An illustration of the experimental protocol is provided in Figure 1.

### Visuomotor Reaction Task

**Figure 2.**
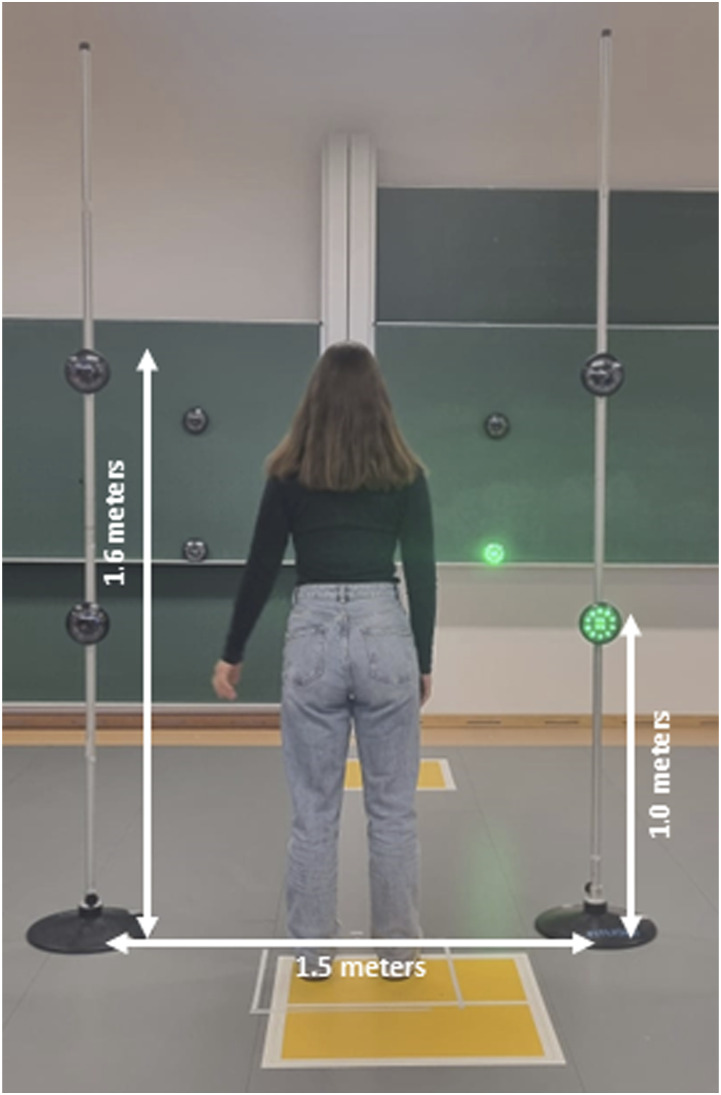
Illustration of the Visuomotor Reaction Task Setup. Fitlight Approximation Sensors Were Arranged in a Rectangular configuration 1.5 meters Apart at Heights of 1 meter (Lower Lights) and 1.6 meters (Upper Lights), Respectively. A Second Set of Synchronized Lights Positioned 2 meters away on a Blackboard Served as Decoy Signals

For the assessment of sports-generic visuomotor RT, Fitlights were used (Sport Corp., Ontario, Canada) using the application for mobile devices (App Version 2.5.7a) with the Wifi Light System (Version 1.31). This system has previously shown high reliability in visuomotor RT assessment (Smith et al., 2024; Čoh et al., 2018). Participants were instructed to stand in a relaxed posture with their arms hanging naturally at their sides and to respond as quickly as possible to LED sensors that lit up in a random sequence. The sensors were equipped with an approximation sensor (sensitivity set to ∼80 cm), allowing for the measurement of visuomotor RT. In total, each trial consisted of 15 lights, with a fixed delay time of 2 seconds between lights. The sensors were arranged in a rectangular configuration and mounted on two vertical bars 1.5 meters apart. The lower two lights in this setup were positioned at a height of 1 meter, while the upper two lights were placed at a height of 1.6 meters. Additionally, a second set of synchronized lights was installed on a blackboard, positioned 2 meters away from the rectangular arrangement. These secondary lights acted as decoy signals, helping to ensure that participants could accurately identify the response lights, even when the lights extended beyond their peripheral vision. The centrally positioned decoy lights also served to control for the slightly reduced peripheral visual field caused by the glasses’ frame in the stroboscopic conditions RTs were recorded and stored through a manufacturer app provided by the FITLIGHT company (App Version 2.5.7a). An illustration of the task setup is provided in Figure 2.

### Statistical Analysis

For the analysis of main-effects of the factors stroboscopic frequency and session day on visuomotor RT, a two-factorial repeated measures analysis of variance (RM-ANOVA) was conducted (SPSS). Strobe frequency (NORMAL, FAST, MEDIUM, SLOW) and session day (I, II, III) served as independent within-subject factors. For each set, the median RT was detected. As each condition was performed twice per session, the trial with the lowest median RT for each condition was used as the dependent variable for that session:. Although some observations were not normally distributed, a RM-ANOVA was applied for analysis, since it has been shown to be robust against violations of the normality assumption (Blanca et al., 2023). In case of violations of sphericity (<.05 for the factor frequency), the Greenhouse-Geisser Correction was applied. The significance level was set to *p* < .05. Bonferroni correction was applied to account for multiple comparisons.

For the analysis of reliability between the testing days, we ran customized scripts for MATLAB (Version R2023a, The Mathworks, Massachusetts, United States). Relative reliability was calculated based on the Intraclass-Correlation-Coefficients (ICC) using a two-way mixed effects model, single ratings and absolute agreement between tests days I, II, and III (Type 3,1). ICC scores were classified as follows: poor (<0.50), moderate (0.50 – 0.75), good (0.75 – 0.90), and excellent (≥0.90) reliability (Koo & Li, 2016). Further, the coefficient of variation (CoV) was calculated as an index of absolute reliability. The CoV is quantified by the ratio between the standard deviation of RTs and the mean RT in the corresponding condition and describes the extent of variability relative to the mean. CoVs <5% were classified as acceptable (Atkinson & Nevill, 1998; Hopkins, 2000).

## Results

The RM-ANOVA revealed a significant main effect of]strobe frequency (*F* (_3,21_) = 174.31; *p* < .001, η_p2_ = .897) on median visuomotor RT. Post-hoc analyses revealed that RTs differed between all conditions and increased stepwise from the NORMAL condition to the FAST, MEDIUM, and SLOW conditions, respectively (p < .001)

**Figure 3.**
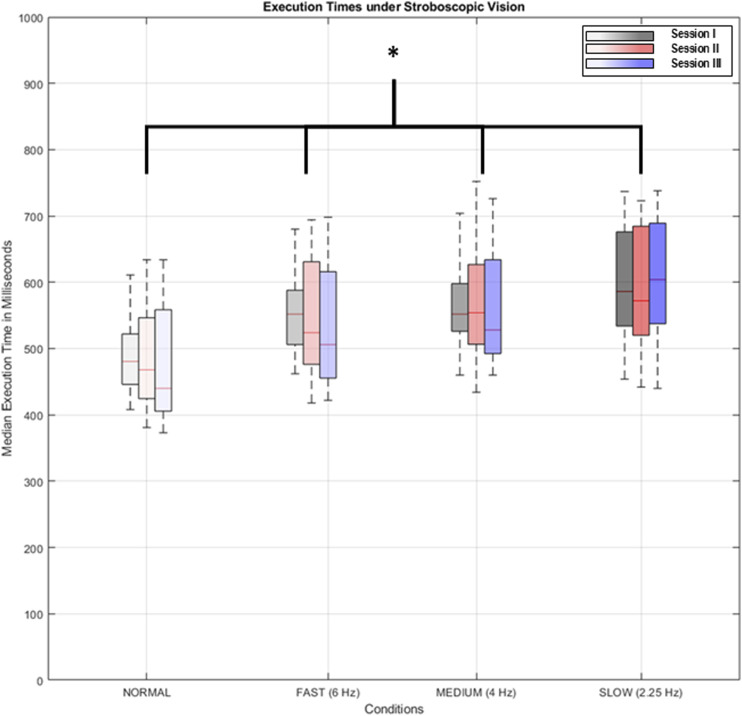
Boxplot of Visuomotor Response Times (RTs) Under Different Visual Conditions (NORMAL, FAST, MEDIUM, and SLOW). Tests Were Repeated Across Three Weekly Sessions. Greyboxes Represent Session I, Red Boxes Represent Session II and Blue Boxes Represent Session III Across the Four Visual Conditions. Dashed Lines Represent the Range From Minimum to Maximum Values in each Condition and Session.* Indicate Significant Differences Across Conditions Revealed by Post-hoc Comparisons (*p* < .05)

No significant main effect has been observed for session day (*F* (_2,21_) = .95; *p* = .395, η_p2_ = .045) and no significant frequency × session day interaction was found (*F* (_6,21_) = 1.95; *p* = .141, η_p2_ = .089), respectively. A boxplot of RTs across different strobe frequencies and session days including significance markers is presented in Figure 3.

**Figure 4.**
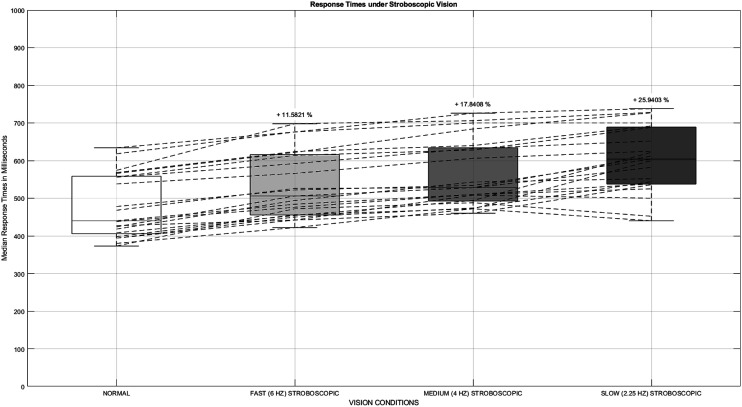
Box- and Lineplot of Inter-individual Differences in Visuomotor RTs Under Different Visual Conditions, including Normal Vision as Well as Fast, Medium and Slow Stroboscopic Vision. The Dashed Lines Display the Individual Responses From the 21 Healthy Adults in the First Experimental Session

Descriptive analyses of individual responses to stroboscopic vision revealed substantial inter-individual variability in responses. Absolute performance decrements from normal to stroboscopic vision ranged from 28 to 90 ms in the fast strobe condition and from 60 to 191 ms in the slow strobe condition. A visualization of the individual changes in RT from NORMAL to SLOW is provided in Figure 4.

### Reliability

Reliability analysis revealed moderate to excellent relative reliability scores for visuomotor RTs with and without stroboscopic vision. Descriptively, ICC scores were higher for the comparison of the second two sessions (Session II vs. Session III) than for the first two sessions (Session I vs. Session II) across all investigated frequencies. In addition, a reduced ICC score was observed for the SLOW stroboscopic condition.

Absolute reliability analysis revealed acceptable CoV scores (CoV <5 %) for all conditions except for the SLOW strobe frequencies. Again, better reliability scores were observed for the comparison of the second two measurement days. An overview of session means, ICC scores and CoV scores is provided in Table 1.

**Table 1.** Overview of Mean Visuomotor Response Times (RTs) Across the Different Stroboscopic Conditions and Testing Sessions. Visuomotor RTs Were Assessed Without Stroboscopic Vision (NORMAL), at Fast (6 Hz), Medium (4 Hz), and Slow (2.25 Hz) Stroboscopic Vision. Intraclass-Correlation-Coefficients (ICCs) Were Computed as Indices of Relative Reliability Using a Two-way Mixed Effects Model, Single Ratings and Absolute Agreement Between Tests Days. The Coefficient of Variation (CoV) was Assessed as a Measure of Absolute Reliability. Lower Bounds (LB) and Upper Bounds (UB) Were Calculated as the 95 % Confidence Interval at an Alpha Level of 0.05

												ANOVA session		
		Response time (ms)			ICC [LB UB]				CoV [LB UB]			F		p
**NORMAL**	**Session I**	507.00	±	64.19	I vs. II	0.88	[0.73	0.95]	5.07	[7.63	3.29]	15.66	0.27		
	**Session II**	494.35	±	88.81											
	**Session III**	487.65	±	91.35	II vs. III	0.95	[0.89	0.98]	3.65	[5.64	2.34]	43.21	0.33		
**FAST**	**Session I**	568.94	±	69.66	I vs. II	0.89	[0.74	0.95]	4.61	[6.95	2.98]	16.86	0.50		
	**Session II**	554.06	±	93.03											
	**Session III**	541.65	±	96.55	II vs. III	0.94	[0.85	0.97]	4.06	[6.23	2.61]	30.21	0.25		
**MEDIUM**	**Session I**	578.53	±	69.77	I vs. II	0.88	[0.73	0.95]	4.55	[6.85	2.95]	15.82	0.70		
	**Session II**	576.65	±	88.48											
	**Session III**	572.24	±	96.27	II vs. III	0.89	[0.75	0.95]	4.98	[7.51	3.22]	17.00	0.27		
**SLOW**	**Session I**	605.06	±	82.43	I vs. II	0.52	[0.12	0.77]	9.86	[13.30	6.79]	3.12	0.89		
	**Session II**	592.59	±	95.09											
	**Session III**	609.65	±	96.47	II vs. III	0.75	[0.47	0.89]	7.71	[11.12	5.11]	6.90	0.96		

## Discussion

The present study aimed to (i) investigate the intra-individual effect of different stroboscopic difficulties on visuomotor RT and (ii) assess the between-day reliability of visuomotor RTs under these conditions. First, we observed a main effect of stroboscopic difficulty, as indicated by a stepwise increase in visuomotor RT following a reduction of the strobe frequency. Second, we observed good to excellent between-day reliability for visuomotor RT under different strobe conditions. However, this reliability decreased at the most difficult setting (i.e., the lowest shutter frequency). Together, these findings suggest that modulations of strobe frequency and duty cycle allow for the systematic and reliable manipulation of visuomotor response times.

As hypothesized, our data reveal a systematic effect of the stroboscopic frequency on visuomotor response time, indicating that slower frequencies and longer duty cycles lead to slower response times across individuals. Performance decreased by 13%, 15%, and 20% under fast (6 Hz), medium (4 Hz), and slow (2.25 Hz) stroboscopic conditions, respectively. Since the reduction of available visual information is the key mechanism underlying stroboscopic training, the settings of frequency and duty cycle determine how much information is removed and allow for the control of task difficulty (Das et al., 2023). Hülsdünker et al. (2019) reported that increased response times under stroboscopic conditions are accompanied by delayed and attenuated processing of information in the visual cortex. Therefore, increased response times may serve as a proxy for this delayed neural processing. Accordingly, it appears that slower stroboscopic frequencies and longer duty cycles challenge the brain and lead to dampened visuomotor performance.

Our observations are in line with findings from cognitive (Bennett et al., 2018) and postural tasks (Kroll et al., 2023; Robey et al., 2024) and underscore the modulatory role of stroboscopic frequencies on task performance. Comparing the absolute effect sizes (partial eta^2^ = .89) in the present study with those presented by Robey et al. (2024) during postural tasks (partial eta^2^ <.75), we speculate that the dose-response-relationship between stroboscopic frequency and visuomotor performance is task-dependent. Particularly in tasks that require quick processing of information such as smash defenses in racket sports (Hülsdünker et al., 2019) or saves in soccer goalkeeping (Wilkins et al., 2018), the duration of occlusion might be more critical than in equilibrium tasks such as balance. However, further comparative analyses investigating the effects of specific strobe settings on different types of tasks are required.

Our between-day comparison of visuomotor RTs under different stroboscopic conditions yielded two interesting findings. First, we observed neither main- nor interaction-effects for the factor of session day. This suggests an absence of short-term learning effects. This observation leads to the assumption that the manipulation of vision through stroboscopic glasses is a reproducible training stimulus. Previous single-session observations have shown that stroboscopic vision slows information processing and delays response times (Hülsdünker et al., 2023). Based on the present findings, it can be anticipated that the detrimental effects of stroboscopic vision on performance remain consistent across multiple occasions, making stroboscopic training a sustainable mid-to long-termapproach to challenge the visual system (Lochhead et al., 2024).

Secondly, we found moderate to excellent relative reliability scores and acceptable absolute reliability scores for visuomotor performance under stroboscopic vision. Brinkman et al. (2021) observed similar reliability scores (.86) for a visuomotor reaction task in a week-to-week analysis but did not incorporate stroboscopic conditions (Brinkman et al., 2021). Therefore, our study reveals that the assessment of visuomotor RT through LED-approximation sensors is reliable, and so are the performance costs induced by stroboscopic vision (Bennett et al., 2018; Robey et al., 2024). In line with our hypothesis, reliability scores were the lowest for the most difficult stroboscopic condition. In the slow frequency, long duty cycle condition, the goggles remained opaque for around 344 ms. Since the glasses and LED sensors were not synced, the relative timing between stimulus presentation and the opaque period varied by trial. When the opaque state appears at random intervals from the LED onset, the potential for error and delayed vision may be greater at low frequencies than at high frequencies. This issue could inflate the trial-to-trial variability within subjects during an experiment. While the limited reliability should be considered when testing visuomotor skills under difficult stroboscopic conditions as proposed for return-to-play testing (Wohl et al., 2021), it is not problematic for training since it reflects visual conditions in the real world. Taken together, both (i) the lack of short-term learning effects and (ii) the high absolute and relative reliability scores of visuomotor RTs under strobe conditions support the use of stroboscopic eyewear to train the visual system.

A secondary finding of our analysis was the marked inter-individual variability in response to increasing strobe difficulty. Performance drops in the slow strobe condition ranged from 60 ms (11% of baseline RT) to 191 ms (42%), indicating that some individuals were more affected by stroboscopic vision than others. While previous stroboscopic studies rarely addressed responder variability, research has linked visual reliance to factors like age and sex (Franz et al., 2015; Ingel et al., 2021). Although these findings stem from postural tasks, similar patterns may underlie visuomotor performance. For instance, Heuvelmans et al. (2023) reported that some soccer players maintained dribbling and passing performance despite 3 Hz visual perturbations. (Heuvelmans et al., 2023). Thus, stroboscopic training induces reliable performance costs that nonetheless vary strongly between individuals.

This point is especially relevant for intervention studies, which often apply fixed progressions of strobe frequency and duty cycle without accounting for inter-individual differences in visual reliance (Das et al., 2023). Our findings suggest that such fixed approaches risk under- or over-challenging participants. We therefore recommend more individualized progression models based on an athlete’s performance, as proposed by Appelbaum et al. (2011), who advanced participants only after reaching specific performance milestones (e.g., five consecutive successful catches) (Appelbaum et al., 2011). For reaction-based tasks, such performance milestones could be based on visuomotor RT reductions falling below a certain threshold (e.g. 15% of baseline), similar to adaptive staircase methods in inhibition training (Verbruggen & Logan, 2009). This individualized approach could help avoid adverse effects like dizziness (Kim et al., 2021), while maintaining an appropriate challenge level. Future studies should consider adopting performance-based progression to identify optimal task difficulty (Guadagnoli & Lee, 2004).

### Limitations

Although the present study is the first to investigate the reliability of visuomotor performance under varying stroboscopic vision conditions, it does not come without limitations. Because of the intra-individual comparisons performed, we did not adjust the visuomotor RT task for arm length & body size. Thus, large individuals had to perform relatively smaller reaching movements in comparison to smaller ones. As this study was a within-subject design, this effect was likely negligible but may have increased the performance variability in the group. Future studies should consider similar motor demands for all individuals when developing visuomotor tasks (Brinkman et al., 2021). Further, the visuomotor RT task performed was sports-generic and due to the simplicity of the task, the number of errors did not vary across stroboscopic frequencies (about 0.2 errors per trial of 15 responses). Due to this clear ceiling-effect, we did not include error counts into our analyses. Since stroboscopic training is also known to influence precision of hand-eye coordination (Ellison et al., 2020), future studies should also investigate visuomotor accuracy across frequencies as a relevant performance outcome. For an effective, performance-based individualization of stroboscopic difficulty, both the change in accuracy and RT should be considered as criteria. Further, inverse efficiency scores that integrate speed and precision into one score could also be promising parameters for a staircase adaptation of strobe settings. In addition, it should be considered that the present study was performed in a sample of young, physically active individuals. Given that stroboscopic glasses are also being explored for orthopedic and neurological rehab, these findings should be applied with caution. Accordingly, a generalization of the reliability of stroboscopic training to specific populations such as the elderly or patients should be avoided (Das et al., 2023). Finally, although there was a clear relationship between strobe frequencies and RTs, subjects reported that the intense light stimulus was still visible through the opaque lenses. Therefore, the impact of slower frequencies on visuomotor performance might have been underestimated and may be augmented when implementing more naturalistic visual stimuli, such as thrown balls or other moving objects (M. Zwierko et al., 2023).

## Conclusion

Overall, this study highlights new implications for stroboscopic training. Firstly, adjusting the difficulty of stroboscopic vision modulates response times, so that the manipulation of strobe difficulty is a simple approach to individualize cognitive demand during training (Perrey, 2022). Further, performance costs induced by stroboscopic training are reliable across three weeks and demonstrate that stroboscopic vision is a stable training stimulus. Together, our data support the application of stroboscopic glasses as a reliable and adaptive training tool allowing for ecologically valid visual training scenarios (Chang et al., 2022). Based on the observed inter-individual variations in strobe-induced performance decrements, a performance-based increase in difficulty appears promising for the optimal use of stroboscopic glasses. Future studies should investigate potential reasons for the differential responsiveness to stroboscopic vision.

## Data Availability

The data that supports the findings of this study are available from the corresponding author upon reasonable request.*
